# Effect of Exogenous Fetuin-A on TGF-*β*/Smad Signaling in Hepatic Stellate Cells

**DOI:** 10.1155/2016/8462615

**Published:** 2016-11-20

**Authors:** Yulai Zhou, Shuang Yang, Pan Zhang

**Affiliations:** ^1^Xiangya School of Medicine, Central South University, Changsha, Huna, 410013, China; ^2^Department of Infectious Diseases, The Third Affiliated Hospital of Xiangya, Central South University, Changsha, Hunan 410013, China

## Abstract

*Objective.* To explore the effects of low concentration of exogenous fetuin-A intervention on TGF-*β*1 induced LX2 cells through detection of the expression of mRNA and protein of Smad2, Smad3, and Smad7.* Methods.* MTT assay was used to detect the LX2 cells proliferation and the regression equation calculating software was applied to determine IC_50_ of fetuin-A. RT-PCR was used to determine the relative content of Smad2, Smad3, and Smad7 mRNA in LX2 cells. Western blot was used to detect the LX2 cells relative content of Smad2, Smad3, Smad7 protein expression, respectively.* Results.* The analysis from RT-PCR and western blot showed that when compared with the other groups TGF-*β*1 + fetuin-A group increased the expression of Smad2 and Smad3 while decreased the expression of Smad7 (*P* < 0.05).* Conclusion.* Fetuin-A may improve the excessive activation of hepatic stellate cells which is caused by an enhanced positive regulation of Smad2 and Smad3 protein and the deficiency in negative regulation of Smad7 protein. This is through inhibiting the expression of Smad2 and Smad3 gene and promoting the expression of Smad7 gene. As a result, the development of liver fibrosis will be reduced.

## 1. Introduction

Fetuin-A, discovered in 1944, is a 59 kDa glycoprotein [1]. It is mainly synthesized by hepatic stellate cells (HSC) and thus closely related to liver. It works as a rare negative acute phase protein, downregulating the activity of macrophages, and has a strong anti-inflammatory effect [2]. Inflammation is one of the major factors that leads to liver fibrosis; thus anti-inflammatory effect of fetuin-A may influence the progress of hepatic fibrosis. At the same time, fetuin-A is also known as a natural TGF-*β* antagonist [3] and is closely associated with TGF-*β*/Smad signaling pathway, which plays a key role in the process of liver fibrosis. Therefore, we inferred that fetuin-A may inhibit the process of liver fibrosis through TGF-*β*/Smad signaling pathway.

## 2. Materials and Methods

### 2.1. Materials

#### 2.1.1. Cultivation of Human Hepatic Stellate (LX2) Cell Line

Human hepatic stellate (LX2) cell lines were obtained from Xiangya central laboratory of Central South University. Cells were cultured in Dulbecco's Modified Eagle Medium (DMEM), which is a modification of Basal Medium Eagle (BME), with a higher concentration of amino acids and vitamins than BME and additional supplemental components. It is also supplemented with 10% fetal bovine serum, 100 U/mL of penicillin, and 100 *μ*g/mL of streptomycin.

*Conditions.* The plates were cultured in a 5% CO_2_ and 100% humidity cell culture box. 

*Reagents.* Trizol, Invitrogen, #15596-026; RevertAid™ H Minus First Strand cDNA Synthesis Kit, Fermentas #K1631; Deoxyribonuclease I (DNase I), Fermentas #EN0521 were used.

RiboLock™ Ribonuclease Inhibitor, Fermentas #EO0381; SYBR Green PCR Master Mix, ABI 4309155, were used. 

*First Antigen.* Mouse Smad7 antibody (1 : 800), SANTA, SC-365846; rabbit TGF*β*1 antibody (1 : 400), SANTA, SC-146; rabbit Smad2/3 antibody (1 : 400), CST, #3102; mouse fetuin-A antibody (1 : 800), SANTA, SC-133146; mouse GAPDH antibody (1 : 800), SANTA, SC-365062, were used. 

*Second Antigen.* Goat anti-mouse IgG/HRP (1 : 80000); goat anti-rabbit IgG/HRP (1 : 40000); goat anti-rabbit IgG/HRP (1 : 40000); goat anti-mouse IgG/HRP (1 : 80000); goat anti-mouse IgG/HRP (1 : 80000) were used.

#### 2.1.2. Establishment of Four Experimental Groups

10% FCS + DMEM culture liquid
TGF-*β*1 experimental group: 10% FCS + DMEM culture liquid + final concentration of 5 ng/mL TGF-*β*1
TGF-*β*1 + fetuin-A experimental group: 10% FCS + DMEM culture liquid + final concentration of 5 ng/mL TGF-*β*1 + 10 ng/mL fetuin-A
TGF-*β*1 + asialoglycoprotein + fetuin-A experimental group: 10% FCS + DMEM culture liquid + final concentration of 5 ng/mL TGF-*β*1 + 10 ng/mL fetuin-A treated with asialoglycoprotein

### 2.2. Methods

#### 2.2.1. Determination of Fetuin-A Concentrations Intervention

Cells were added in 96-well microtiter plates (100 *μ*L/hole, approximately 1 × 10^4^) and were cultured at 37°C in a 5% CO_2_ humidified incubator for 24 hours which were then mixed with the appropriate concentration of tested compounds. The plates were cultured in a 5% CO_2_ and 100% humidity cell culture box. Each hole was added with 50 *μ*L 1x MTT and incubated for 4 hours. Discard supernatant, and 150 *μ*L DMSO was added to each hole to dissolve the armour and was shaken. The optical density of each hole at was detected at 570 nm. The temperature will remain the same during the whole process.

#### 2.2.2. RNA Isolation and Purification and Real-Time PCR

Total RNA was extracted from cells using Trizol reagent with the instructions of Invitrogen. Reverse transcription was performed using RevertAid™ H Minus First Strand cDNA Synthesis Kit (Fermentas) according to the manufacturer's protocol. Real-time PCR samples were prepared with SYBR Green PCR Master Mix (ABI 4309155) and real-time PCR was performed with an ABI Prism 7500 Detector System. The Housekeeping Gene GADPH was used as an internal control. The real-time PCR primers were from Gene Bank (BCOl2678, mouse fetuin-A cDNA). Primer sequences are as follows: Smad2 gene (180 bp) upstream primer: 5-cggtagaaatgacaagaagg-3, downstream primer: 5-tcttcagattacagcctggt-3; Smad3 gene (155 bp) upstream primer: 5-gtccagtctcccaactgtaa-3, downstream primer: 5-aactggtagacagcctcaaa-3; Smad7 gene (169 bp) upstream primer: 5-atgatctacctcaggggaat-3, downstream primer: 5-gacttgatgaagatggggta-3; *β*-actin gene (208 bp) upstream primer: 5-cattaaggagaagctgtgct-3, downstream primer: 5-gttgaaggtagtttcgtgga-3.

The real-time PCR system contains a template of 1 *μ*L; Primer A 100 nm; Primer B 100 nm; 2x SYBR Green PCR Master mix 12.5 *μ*L; DDW 25 *μ*L. Parameters are as follows: 94°C 5 min; 94°C 20 s, 61°C 20 s, 72°C 20 s, 40 cycles; 72°C 5 min; 55°C 10 s; +0.5°C/cycle 10 s (80 cycles). The melting curve was analyzed after the amplification (detection between 60–95°C) to determine DEGC. The conditions were set at incremental increase of 0.5°C and 5 s each cycles. After agarose gel electrophoresis and ethidium bromide coloration, the data obtained from the assays were analyzed with eagle eye II gel imaging and analysis system for digital conversion. To show the relative expression of Smad2, Smad3, and Smad7, fold change of expression was then calculated using the 2^−ΔΔCt^ method [4].

#### 2.2.3. Western Blot Analysis

Western blot analysis was conducted according to previous studies [5, 6]. The cellular lysates extracted from the cells were used for protein assays. Protein concentration was detected by a spectrophotometer using a BCA protein assay kit. Equal amounts of protein were subjected to SDS-PAGE on a 10% poly-acrylamide gel and transferred to a polyvinylidene difluoride membrane (Millipore). The membrane with blotted protein was blocked for 1 hour at room temperature with blocking buffer containing 5% BSA, followed by incubation with antibodies overnight at 4°C. GAPDH was used as an internal control. After SDS-PAGE, the proteins were transferred onto the PVDF membrane and hybridized with specific primary antibodies and were incubated with HRP-conjugated sheep anti-mouse IgG. Bands were viewed using the ECL kit (Amersham, Piscataway, NJ) according to the manufacturer's instruction.

#### 2.2.4. Statistical Analysis

The data was presented as a mean ± SD. Using SPSS 15.0 software (SPSS, Chicago, IL), one-way analysis of variance (ANOVA) test was employed for the comparison among all groups. Levene's test was then applied to compare the statistical difference between the two groups. Tamhane's T2 test was used when the variance was unequal. All tests were two-sided and a *P* value < 0.05 was considered to be statistically significant. The comparison of means between groups was made up by ANOVA.

## 3. Results

### 3.1. The Determination of the Interfering Concentration of Fetuin-A

**Figure 1:**
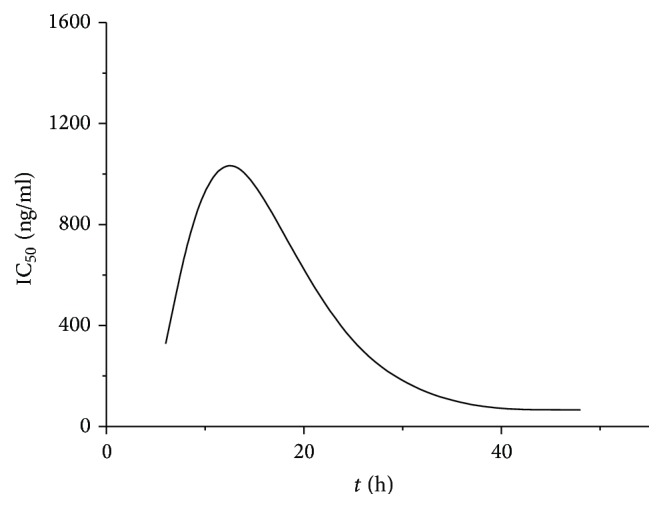
Time-IC_50_ of fetuin-A.

According to the results of MTT Assays (Figure 1), the concentration was set at fetuin-A 10 ng/mL.

### 3.2. Real-Time PCR

**Figure 2:**
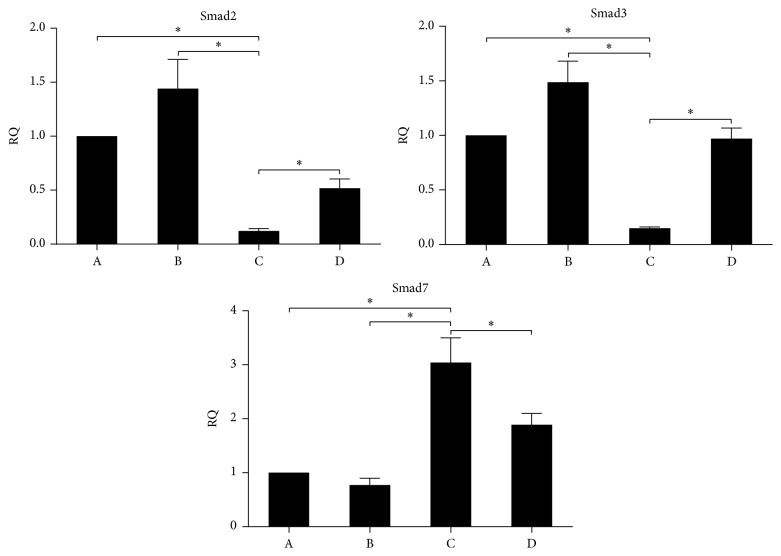
Relative expression of Smad2, Smad3, and Smad7. A: blank control group; B: TGF-*β*1 experimental group; C: TGF-*β*1 + fetuin-A experimental group; D: TGF-*β*1 + asialofetuin-A experimental group. The relative expression of Smad2, Smad3, and Smad7 was analyzed by RT-PCR. Experimental treatments were analyzed in triplicate. Data were represented as mean ± SD. Statistics were analyzed with a one-way analysis of variance (ANOVA) test. Asterisk represents *P* < 0.05.

To monitor for endogenous fetuin-A retention and exogenous fetuin-A supplement, the RQ value of each group were measured. Statistical analysis (Figure 2) suggests that the gene transcription of Smad2 in samples in group C was lower than that of the samples in groups A, B, and D. Both groups A and D were negative control groups (*P* < 0.0001). The values of group C were significantly lower than group B sample (0.123 ± 2.02% versus 1.441 ± 2.70%). The analysis of Smad3 shows similar results, whereas the results for Smad7 were conversely represented. In summary, mRNA expressions of Smad2 and Smad3 in group C were decreased, while Smad7 was increased. The differences were all statistically significant.

### 3.3. Western Blot

**Figure 3:**
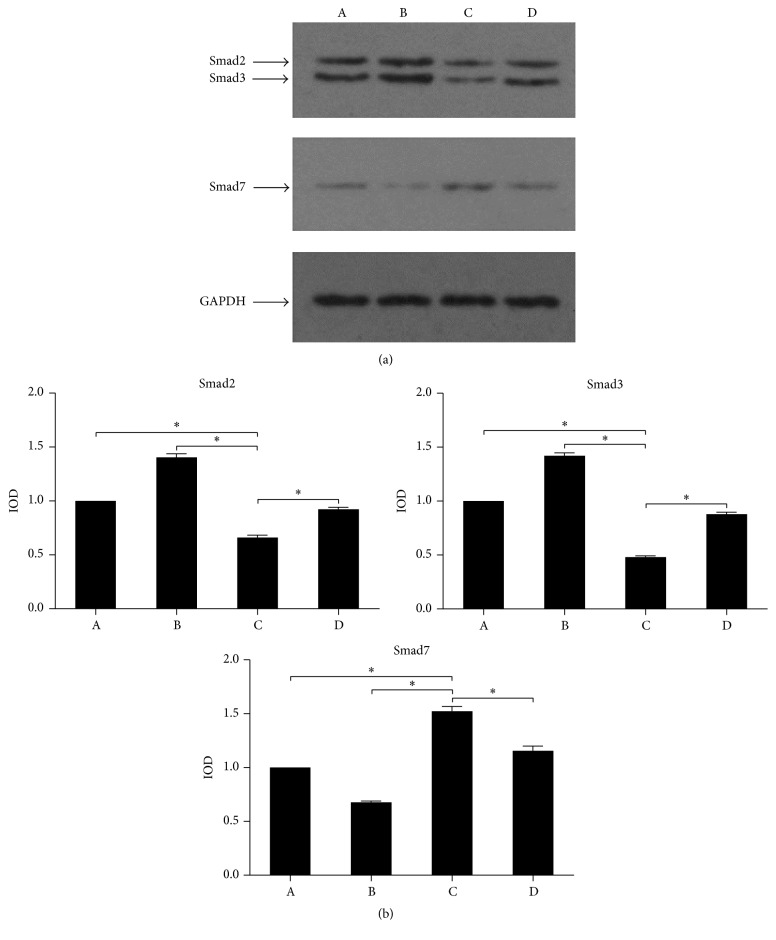
The protein abundance of Smad2, Smad3, and Smad7. (a) Representative bands showing the changes of Smad2, Smad3, Smad7, and GAPDH. Lane A: blank control group; Lane B: TGF-*β*1 experimental group; Lane C: TGF-*β*1 + fetuin-A experimental group; Lane D: TGF-*β*1+ asialofetuin-A experimental group. (b) Densitometric analysis of Smad2, Smad3, Smad7, and GAPDH. Relative protein expression was analyzed by western blot. Experimental treatments were analyzed in quadruplicate. The positive bands were quantitatively analyzed by Gel Pro 4.0 Analysis software after their integrated optical density (IOD) was measured. The housekeeping gene (GADPH) was used as an internal control. Data were represented as mean ± SD. Statistics were obtained with a one-way analysis of variance (ANOVA) test. Asterisk represents *P* < 0.05.

To confirm well established functional consequence of exogenous fetuin-A, we analyzed differences in the translation of Smad2, Smad3, and Smad7 genes involved in TGF-*β*/Smad signaling pathway. The results of western blot (Figure 3) determine that mRNA translation of Smad2 and Smad3 in group C was significantly downregulated (0.662 ± 2.03% versus 1.404 ± 3.36%, 0.481 ± 1.19% versus 1.421 ± 2.65%), while Smad7 was significantly increased (1.522 ± 4.53% versus 0.677 ± 1.23%). The differences showed statistical significance. The housekeeping gene GADPH was used as an internal control and the differences between each experimental group were not statistically significant.

## 4. Discussion

Liver fibrosis is the excessive accumulation of extracellular matrix proteins, such as collagen. It is considered as a wound healing response to chronic liver injury. It always indicates the onset of progressive disease, which may eventually lead to cirrhosis and end-stage liver disease [7, 8]. HSC has a major role in the accumulation of extracellular matrix (ECM) which contributes to fibrogenesis. This is activated mainly through TGF-*β*/Smad pathway. Therefore, this experiment has attempted to explore how fetuin-A affects the gene expression of Smad2, Smad3, and Smad7, researching for how fetuin-A affects the pathway as well as its potential influence on HSC activation.

TGF-*β* works through multiple signaling pathways, affecting cell proliferation, apoptosis, recession, differentiation, and migration [9]. It is known as a major cytokine, with a complex function in HSC activation accelerating liver fibrosis [10, 11]. Its signaling pathways include Smad dependent and Smad independent pathways. Recent studies show that Smad proteins are important substrate for TGF-*β* receptor intracellular kinase. Furthermore, they effectively mediated in the TGF-*β* in the intracellular signal transduction [12, 13].

The classical TGF-*β*/Smad pathway is a highly conserved linear cascade process. Firstly, TGF-*β* was combined with TGF*β* type II receptor (TGF*β*RII) on cell membrane, providing it with kinase activity, and then combined with TGF*β*I receptor (TGF*β*RI). Activated TGF*β*RI was then combined with restriction Smad (R-Smad), phosphorylating the COOH end of Smad2 or Smad3. This formed R-S heteromeric transcription complexes with Smad4 which shifted towards the nucleus and bonded with sequence specific DNA. This complex plays a role in transcriptional regulation [14], mediating HSC transforming to fibroblast (myofibroblaster, MFB) and leading to the development of fibrosis [15–18]. Smad7 gene is an inhibitor of TGF-*β* signaling pathway (inhibition Smad, I-Smad). Smad7 inhibits the phosphorylation of Smad2 and Smad3 through the activation of TGF*β*RI, while inhibiting Smad2 and Smad3 combination with receptors. This formed a negative feedback loop in TGF-*β* signaling and effectively antagonizied fibrogenesis [19, 20]. On the other hand, I-Smad can enhance the interaction of E3 ubiquitin protein ligase, Smad ubiquitin regulatory factor 1/2 (Smurf 1/2) and receptor complex, to regulate receptor update [21, 22].

When liver injury occurs, TGF-*β* enables HSC activation, proliferation, and differentiation into MFB, promoting collagen production. TGF-*β* mediates wound healing response through ALK/Smad2 and Smad3 signaling pathway [23]. The ongoing presence of the damaging factors alters the phenotypic conversion of HSC. As a result, the inhibition effect of Smad7 on the TGF-*β* negative feedback will be reduced. Consequently, HSC is excessively activated, causing the ECM metabolic imbalance. Eventually, the progression of liver fibrosis is deteriorated [24, 25]. Compared with the blank control group, the gene expression of Smad2 and Smad3 in the TGF-*β*1 experimental group was increased while Smad7 was decreased, indicating that TGF-*β*/Smad pathway may be involved in the process of excessive activation of HSC and hepatic fibrosis.

Fetuin-A is mainly synthesized and secreted by the liver. During the fetal period, the concentration of fetuin-A in serum is high and drops significantly after an inflammatory injury [26–28]. Fetuin-A is also a natural TGF-*β* antagonist [29], which can be applied in the TGF-*β*/Smad signaling pathway. Fetuin-A and TGF*β*RII have 18~19 amino acid sequence in common. Fetuin-A can be combined with TGF-*β* related cytokines such as bone morphogenetic proteins 2, 4, and 6 and TGF-*β*1 and TGF-*β*2, thus competitively blocking the combination of TGF-*β* and its TGF*β*RII on the cell surface. A study conducted by Carol confirmed that fetuin-A can inhibit the combination of TGF-*β* and its receptor and thus blocking the phosphorylation and nuclear translocation of Smad2 and Smad3 [30]. Judging from the close relationship between TGF-*β*/Smad signaling pathway and liver fibrosis, we can speculate that fetuin-A has the potential to affect the progression of liver fibrosis via the TGF-*β*/Smad signaling pathway.

MTT assay was used to detect the proliferation of LX2 cells. The half inhibitory concentration (IC_50_) of fetuin-A was then calculated by the regression equation software. The optimal concentration of fetuin-A was 10 ng/mL (Figure 1). At this concentration, the results of RT-PCR (Figure 2) and western blot (Figure 3) showed that, in comparison to the other groups, the gene expression of Smad2 and Smad3 in TGF-*β*1 + fetuin-A experimental group was enhanced while expression of Smad7 was suppressed. These differences were all statistically significant. Fetuin-A may reduce HSC hyperactivity and the severity of hepatic fibrosis. This is achieved by the suppression of Smad2 and Smad3 gene expression whereas the gene expression of Smad7 was promoted.

In conclusion, fetuin-A could be a protective agent for hepatic fibrogenesis through the TGF-*β*/Smad signaling pathway. Supplementation with exogenous fetuin-A could alter the balance between inflammation and liver fibrosis by reducing the inflammatory effect. Given that fetuin-A represents biological homology, a high affinity, and no significant side effects, the administration of fetuin-A confers protection against hepatic fibrosis. Therefore, further studies are required to explore the therapeutic potential of fetuin-A in the clinical management of acute liver failure.

## References

[1] Pedersen K. O. (1944). Fetuin, a new globulin isolated from serum. *Nature*.

[2] Wang H., Li W., Zhu S., Wang P., Sama A. E., Veas F. (2011). Role of fetuin-A in injury and infection. *Acute Phase Proteins—Regulation and Functions of Acute Phase Proteins*.

[3] Dai X.-H., Zhang P., Xiao M.-F. (2011). Protective role of *α*2HS-glycoprotein in HBV-associated liver failure. *International Journal of Molecular Sciences*.

[4] Livak K. J., Schmittgen T. D. (2001). Analysis of relative gene expression data using real-time quantitative PCR and the 2^−∆∆*c*_T_^ method. *Methods*.

[5] Ren W., Chen S., Yin J. (2014). Dietary arginine supplementation of mice alters the microbial population and activates intestinal innate immunity. *The Journal of Nutrition*.

[6] Ren W., Yin J., Wu M. (2014). Serum amino acids profile and the beneficial effects of L-arginine or L-glutamine supplementation in dextran sulfate sodium colitis. *PLoS ONE*.

[7] Koller T., Kollerova J., Huorka M., Meciarova I., Payer J. (2014). Noninvasive scoring algorithm to identify significant liver fibrosis among treatment-naive chronic hepatitis C patients. *European Journal of Gastroenterology and Hepatology*.

[8] Fuchs B. C., Hoshida Y., Fujii T. (2014). Epidermal growth factor receptor inhibition attenuates liver fibrosis and development of hepatocellular carcinoma. *Hepatology*.

[9] Wu M., Xiao H., Liu G. (2016). Glutamine promotes intestinal SIgA secretion through intestinal microbiota and IL-13. *Molecular Nutrition & Food Research*.

[10] Gressner A. M., Weiskirchen R. (2006). Modern pathogenetic concepts of liver fibrosis suggest stellate cells and TGF-*β* as major players and therapeutic targets. *Journal of Cellular and Molecular Medicine*.

[11] Presser L. D., McRae S., Waris G. (2013). Activation of TGF-*β*1 promoter by hepatitis C virus-induced AP-1 and Sp1: role of TGF-*β*1 in hepatic stellate cell activation and invasion. *PLoS ONE*.

[12] Ding Z.-Y., Jin G.-N., Liang H.-F. (2013). Transforming growth factor *β* induces expression of connective tissue growth factor in hepatic progenitor cells through Smad independent signaling. *Cellular Signalling*.

[13] Matsuzaki K. (2013). Smad phospho-isoforms direct context-dependent TGF-*β* signaling. *Cytokine and Growth Factor Reviews*.

[14] Moon S.-K., Kim W.-J., Choi Y. H. (2014). Melittin inhibits TGF-*β*-induced pro-fibrotic gene expression through the suppression of the TGF*β*RII-Smad, ERK1/2 and JNK-mediated signaling pathway. *The American Journal of Chinese Medicine*.

[15] Dooley S., Ten Dijke P. (2012). TGF-*β* in progression of liver disease. *Cell and Tissue Research*.

[16] Paradis V., Dargere D., Bonvoust F., Vidaud M., Segarini P., Bedossa P. (2002). Effects and regulation of connective tissue growth factor on hepatic stellate cells. *Laboratory Investigation*.

[17] Long J., Wang G., Matsuura I., He D., Liu F. (2004). Activation of Smad transcriptional activity by protein inhibitor of activated STAT3 (PIAS3). *Proceedings of the National Academy of Sciences of the United States of America*.

[18] Yoshida K., Matsuzaki K. (2012). Differential regulation of TGF-*β*/Smad signaling in hepatic stellate cells between acute and chronic liver injuries. *Frontiers in Physiology*.

[19] Goumans M. J., Mummery C. (2002). Functional analysis of the TGF *β* receptor/Smad pathway through gene ablation in mice. *International Journal of Developmental Biology*.

[20] Taylor I. W., Wrana J. L. (2008). SnapShot: the TGF*β* pathway interactome. *Cell*.

[21] Kavsak P., Rasmussen R. K., Causing C. G. (2000). Smad7 binds to Smurf2 to form an E3 ubiquitin ligase that targets the TGF*β* receptor for degradation. *Molecular Cell*.

[22] Dooley S., Hamzavi J., Breitkopf K. (2003). Smad7 prevents activation of hepatic stellate cells and liver fibrosis in rats. *Gastroenterology*.

[23] Seyhan H., Hamzavi J., Wiercinska E. (2006). Liver fibrogenesis due to cholestasis is associated with increased Smad7 expression and Smad3 signaling. *Journal of Cellular and Molecular Medicine*.

[24] Dooley S., Streckert M., Delvoux B., Gressner A. M. (2001). Expression of Smads during in vitro transdifferentiation of hepatic stellate cells to myofibroblasts. *Biochemical and Biophysical Research Communications*.

[25] Dooley S., Delvoux B., Streckert M. (2001). Transforming growth factor *β* signal transduction in hepatic stellate cells via Smad2/3 phosphorylation, a pathway that is abrogated during in vitro progression to myofibroblasts. *FEBS Letters*.

[26] Wang H., Sama A. E. (2012). Anti-inflammatory role of fetuin-A in injury and infection. *Current Molecular Medicine*.

[27] Li W., Zhu S., Li J. (2011). A hepatic protein, fetuin-A, occupies a protective role in lethal systemic inflammation. *PLoS ONE*.

[28] Szweras M., Liu D., Partridge E. A. (2002). *α*2-HS glycoprotein/fetuin, a transforming growth factor-*β*/bone morphogenetic protein antagonist, regulates postnatal bone growth and remodeling. *The Journal of Biological Chemistry*.

[29] Demetriou M., Binkert C., Sukhu B., Tenenbaum H. C., Dennis J. W. (1996). Fetuin/*α*2-HS glycoprotein is a transforming growth factor-*β* type II receptor mimic and cytokine antagonist. *Journal of Biological Chemistry*.

[30] Swallow C. J., Partridge E. A., Macmillan J. C. (2004). *α*2HS-glycoprotein, an antagonist of transforming growth factor *β* in vivo, inhibits intestinal tumor progression. *Cancer Research*.

